# Association between sputum conversion and in-hospital mortality in elderly patients with pulmonary tuberculosis: a retrospective study

**DOI:** 10.1186/s12879-022-07334-1

**Published:** 2022-04-05

**Authors:** Yuta Nakamura, Mari Yamasue, Kosaku Komiya, Shuichi Takikawa, Kazufumi Hiramatsu, Jun-ichi Kadota

**Affiliations:** 1 Internal Medicine, National Hospital Organization Nishi-Beppu Hospital, 4548, Tsurumi, Beppu, Oita 874-0840 Japan; 2 Respiratory Medicine and Infectious Diseases, Oita University Faculty of Medicine, 1-1 Idaigaoka, Hasama-machi, Yufu, Oita 879-5593 Japan; 3 Medical Safety Management, Oita University Faculty of Medicine, 1-1 Idaigaoka, Hasama-machi, Yufu, Oita 879-5593 Japan

**Keywords:** Tuberculosis, Conversion, Elderly, Mortality

## Abstract

**Background:**

Non-conversion of sputum culture or smear within 2 months after the start of treatment is a known poor prognostic factor of pulmonary tuberculosis. In elderly patients, sputum conversion may be delayed because of the age-related decline in immune competence. This study aimed to assess how a long interval to sputum conversion predicts in-hospital mortality in elderly patients with pulmonary tuberculosis.

**Methods:**

Consecutive elderly patients (age > 65 years) who were admitted to our institution for bacteriologically confirmed pulmonary tuberculosis were included. The association between sputum conversion within 30, 60, 90, or 120 days from the start of treatment and in-hospital mortality were analyzed by Cox proportional-hazards regression after adjustment for other potential variables.

**Results:**

This study included 262 patients, and 74 patients (28%) died during hospitalization. Multivariate analyses showed that sputum non-conversion within 90 days (adjusted hazard ratio 0.424, 95% CI 0.252–0.712, p = 0.001) or 120 days (0.333, 0.195–0.570, p < 0.001) was independently associated with in-hospital mortality, whereas that within 60 days was not (p = 0.890).

**Conclusions:**

In elderly patients with tuberculosis, 2 months may be insufficient when evaluating sputum conversion as a prognostic factor. Sputum non-conversion within 90 days or longer may predict in-hospital mortality more accurately.

## Background

While the prevalence of tuberculosis (TB) has gradually declined worldwide, the incidence and mortality of TB in the elderly population is still high in high- and middle-income countries in which the elderly population is increasing [1]. Advanced age, multidrug-resistant TB, malnutrition, activities of daily living, human immunodeficiency virus infection, liver disease, renal disease, diabetes mellitus, and poverty have been reported as prognostic factors in patients with TB [2–6]. In addition, non-conversion of sputum culture or smear within 2 months after the start of treatment is widely recognized as a predictive factor for poor prognosis and treatment failure in patients with pulmonary TB [7–11]. A study showed that patients with negative sputum smears within 2 months after treatment were three times more likely to be successfully treated [12].

However, these studies were mostly published from countries with high TB burden, and the study population was characterized by non-elderly patients. Currently, in many high- and middle-income countries with intermediate TB burden, a substantially high TB incidence is reported among elderly people, reflecting reactivation following latent infection in aging people. TB patients aged ≥ 60 years accounted for 72% of all TB patients in Japan [13] in 2020, and similar patterns have been reported in other countries [14–16]. For example, the TB infection rate among people aged > 85 years is as high as 100 times greater than children aged < 15 years in Hong Kong [15]. Nevertheless, no study has assessed the accurate interval to sputum conversion for determining the prognosis in elderly patients.

We previously reported that sputum conversion within 2 months after the start of treatment was not related to prognosis in elderly patients with pulmonary TB [17]. In elderly patients, sputum conversion may be delayed due to the age-related decline in the immune function. It is unclear if the interval from start of treatment to sputum conversion accurately predicts disease prognosis in elderly patients with pulmonary TB.

We hypothesized that negative conversion may be delayed in elderly patients, and this could contribute to a poor prognosis. However, it is noted that other factors—such as inflammation, nutritional status and physical activity level—may also affect disease progression and are therefore required to be considered as confounders. Our study therefore aimed to determine the impact of delayed conversion to negative on in-hospital mortality and then to assess which interval from the start of treatment to sputum conversion is mostly associated with in-hospital mortality, adjusting for potential confounders in elderly patients with pulmonary TB.

## Methods

### Patients and study design

This was a retrospective cohort study conducted at National Hospital Organization Nishi-Beppu Hospital in Oita Prefecture, Japan. We included consecutive elderly patients (> 65 years old) with bacteriologically confirmed pulmonary TB who were admitted to the hospital between January 2013 and December 2016. The study protocol was approved by the institutional ethics committee of our institution (approval number: 1–4; approval date: September 25, 2019) and followed the Declaration of Helsinki Ethical Principles for medical research involving human subjects. The need for informed consent was waived by the committee because of the retrospective design of the study. Information regarding this research was posted at the hospital, with an opt out method. Some of the subjects included in this study had already participated in previous studies [17–22].

### Data collection and definitions

The following patient data were obtained from the medical records: age, sex, body mass index, physical activity level, underlying diseases, respiratory failure, smoking history, laboratory data, sputum information including smear grade, drug sensitivities, interval between sputum cultures, and date of sputum conversion, and chest computed tomography findings. We evaluated daily physical activity upon admission using a performance status (PS) scale [23]. Respiratory failure was defined as an oxygen saturation of 90% without oxygen therapy upon admission.

We defined sputum conversion as the situation when two consecutive cultures taken at least 30 days apart were found to be negative. In this case, the specimen collection date of the first negative culture was used as the date of conversion in accordance with World Health Organization definitions [24]. However, the Japanese Society of Tuberculosis allows patients with pulmonary TB to be discharged by confirmation if they have three consecutive negative cultures at least 2 weeks after the start of treatment. Thus, patients who were discharged by meeting this criterion without an examination following sputum culture were regarded as censored in this study. The primary outcome was all-cause in-hospital mortality. We classified patients who died in the hospital and those who survived till discharge as non-survivor and survivor groups, respectively. In our hospital, patients are required to discharge after confirmation of three negative results by sputum culture. Therefore, all patients received ongoing anti-TB treatments during hospitalization, and in patients who died in the hospital, we did not confirm negative conversion at the time of death. “In-hospital death” implies non-recovery from TB infection, and does not compete with sputum conversion. However, we may have confirmed some patients as negative conversion after death because it takes 6 weeks to obtain a negative culture result, and these cases were deemed to meet negative conversion in this study.

### Statistical analysis

Statistical analyses were performed using the Statistical Package for the Social Sciences software version 25 (IBM Japan, Tokyo, Japan). The sputum conversion rates within 60, 90, or 120 days from the start of treatment, patient characteristics, and clinical data in the non-survivor group were compared to those in the survivor group. These information are routinely collected on admission for all patients with TB in our hospital.

The time to in-hospital mortality between negative conversions or not within 60, 90, and 120 days, respectively, were compared using the log-rank tests. We considered variables with a p-value of < 0.05 in the univariate analysis as eligible for entry into the multivariate Cox proportional-hazards regression analysis. Since some variables may have collinearity (for instance, albumin and hemoglobin), focusing on more clinically significant factors, we tested the goodness of fit to multivariate analysis for selecting covariates in a stepwise manner. Eventually, we conducted multivariate analyses in three models because sputum conversion rates within 60, 90, and 120 days significantly differed between non-survivors and survivors.

## Results

### Baseline characteristics and conversion rates in the non-survivor and survivor groups

**Fig. 1:**
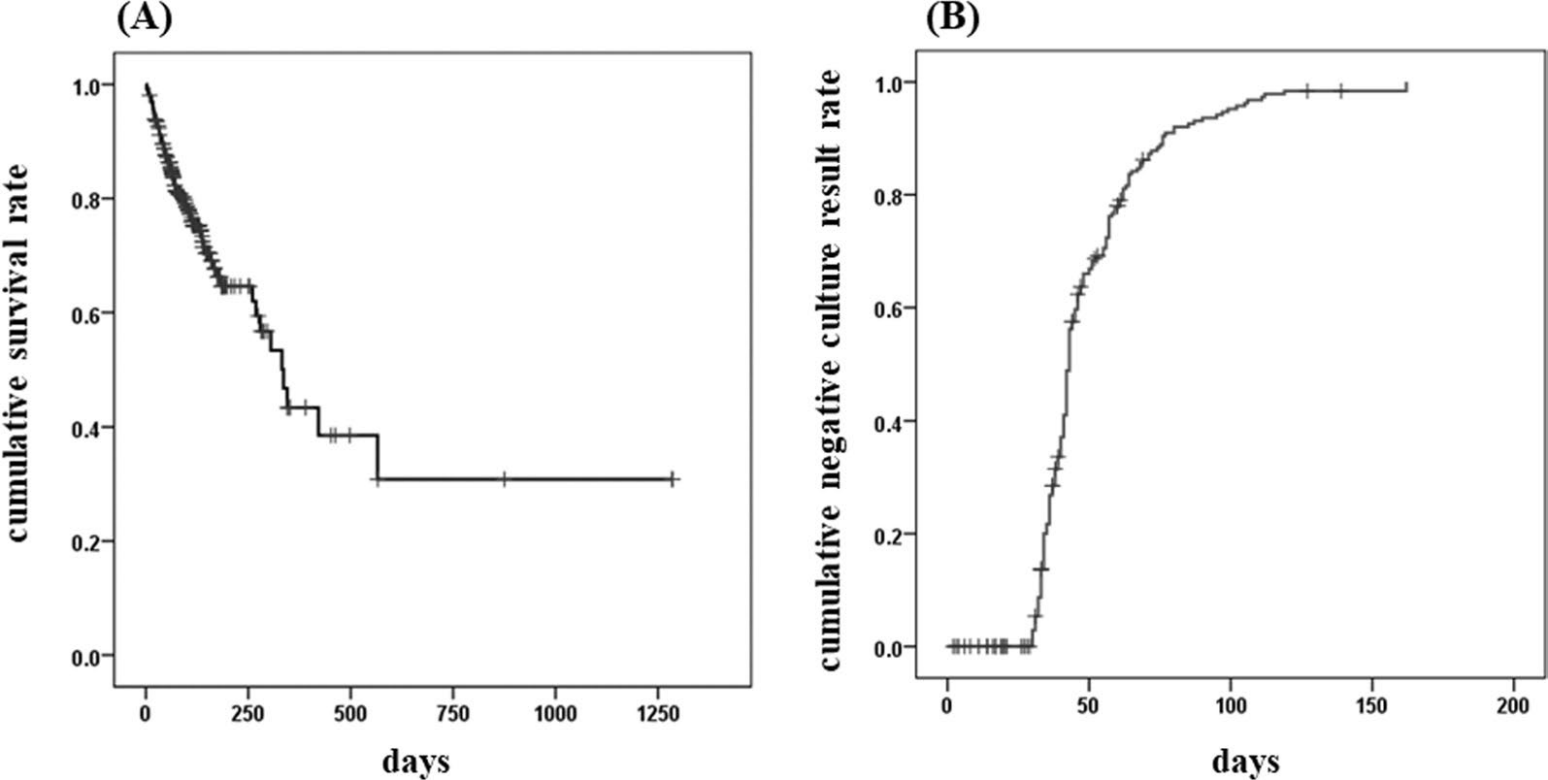
Kaplan–Meier estimate of survival probability (**A**) and positive sputum culture results (**B**) among all included elderly patients with pulmonary tuberculosis

This study included 262 patients with a median age of 84 years, and 74 patients (28%) died during hospitalization. In this study, we included 262 patients with a median age of 84 years, and 74 patients (28%) died during hospitalization. We confirmed 184 of 188 patients (98%) as negative for conversion among the survivors. Of the four remaining patients, one was transferred to another hospital and three were discharged prior to 30 days in accordance with Japanese guidelines, so they were not examined following sputum culture. In contrast, we observed negative conversion in 36 (49%) patients among non-survivors. As mentioned in the "Methods" section, patients are required to discharge after consecutive negative conversions are confirmed in our hospital. Therefore, in these cases, negative conversion was confirmed by the sputum culture submitted before death. In fact, the Kaplan-Meier curve showed that negative conversion was partially reached prior to in-hospital death (Fig. 1).

*Mycobacterium tuberculosis* that was resistant to more than one first-line anti-TB drug was isolated in 16 patients (6%) as follows (overlap permitted): pyrazinamide (n = 1) and streptomycin (n = 1) in the non-survivor group, and isoniazid (n = 7), pyrazinamide (n = 7), ethambutol (n = 1), and streptomycin (n = 4) in the survivor group; resistance to a combination of isoniazid and rifampin was unnoted. We administered combination therapy of four first-line anti-TB drugs as the standard regimen—rifampicin, pyrazinamide, isoniazid, and ethambutol or streptomycin—to 142 patients (54%). Seven patients (10%) among non-survivors and 9 patients (5%) among survivors were treated with pyrazinamide, isoniazid, ethambutol, and levofloxacin, and 3 patients (4%) among non-survivors and 7 patients (4%) among survivors were treated with rifampicin, pyrazinamide, isoniazid, and levofloxacin. The other patients were treated with various combinations of anti-TB drugs. All patients received directly observed treatment. While standard therapy was not associated with conversion rate (131/142, 92% in standard therapy and 102/120, 91% in non-standard therapy, crude hazard ratio 1.003, 95% CI 0.773–1.301, p = 0.983), it was significantly associated with in-hospital mortality in univariate analysis, as shown in Table 1.

**Table 1:** Univariate analysis of the baseline characteristics associated with in-hospital mortality of patients with pulmonary tuberculosis

	Non-survivor n = 74	Survivor n = 188	Crude hazard ratio (95% CI)	*P*
Female	39 (53)	95 (51)	1.131 (0.714–1.792)	0.600
Age, years old	87 (81–90)	82 (78–88)	1.062 (1.023–1.102)	0.002
body mass index, kg/m^2^	17.8 (15.7–20.4)	19.1 (17.4–21.2)	0.931 (0.867–1.000)	0.052
Performance status	4 (3–4)	2 (1–3)	3.007 (2.145–4.217)	< 0.001
Smoker	15 (20)	32 (17)	1.049 (0.594–1.855)	0.869
COPD	7(9)	14 (7)	1.103 (0.500–2.433)	0.808
Heart failure	22 (30)	26 (14)	1.706 (1.033–2.816)	0.037
Cerebrovascular disease	14 (19)	30 (16)	1.392 (0.776–2.497)	0.267
Diabetes mellitus	21 (28)	45 (24)	1.200 (0.722–1.993)	0.481
Chronic kidney disease	17 (23)	18 (10)	2.817 (1.624–4.888)	< 0.001
Hepatic diseases	11 (15)	8 (4)	2.551 (1.341–4.852)	0.004
Respiratory failure	46 (62)	39 (21)	3.525 (2.196–5.659)	< 0.001
Smear grade > 2+	12 (16)	30 (16)	0.632 (0.330–1.208)	0.165
Resistance to first-line drug	2 (3)	14 (7)	0.397 (0.097–1.619)	0.198
Standard therapy	25 (34)	117 (62)	0.373 (0.230–0.605)	< 0.001
Conversion	36 (49)	184 (98)	0.120 (0.075–0.190)	< 0.001
Conversion within 60 days	33 (45)	147 (78)	0.594 (0.359–0.983)	0.043
Conversion within 90 days	35 (47)	175 (93)	0.218 (0.136–0.349)	< 0.001
Conversion within 120 days	36 (49)	183 (97)	0.132 (0.083–0.209)	< 0.001
Average interval of sputum culture (week)	2 (1.5–2)	2 (1.5–2)	1.024 (0.666–1.577)	0.912
WBC (×10^3^/µL)	6.7 (4.7–10.2)	6.5 (5.2–8.1)	1.028 (0.956–1.106)	0.456
C-reactive protein (mg/dL)	6.5 (3.4–11.7)	2.6 (0.7–6.4)	1.046 (1.019–1.073)	0.001
Hemoglobin (g/dL)	10.3 (9.1–11.4)	11.5 (10.2–12.9)	0.702 (0.619–0.796)	< 0.001
Albumin (g/dL)	2.1 (1.7–2.5)	3.0 (2.5–3.5)	0.174 (0.111–0.273)	< 0.001
AST (IU/L)	28 (22–44)	25 (20–35)	1.005 (1.003–1.008)	< 0.001
ALT (IU/L)	18 (12–29)	16 (11–25)	1.007 (1.004–1.010)	< 0.001
BUN (mg/dL)	26.0 (16.8–39.0)	16.5 (12.6–21.2)	1.035 (1.025–1.046)	< 0.001
Creatinine (mg/dL)	0.78 (0.45–1.15)	0.73 (0.56–0.89)	1.666 (1.322–2.100)	< 0.001
Number of lobes	4 (3–5)	3 (2–4)	1.226 (1.037–1.449)	0.017
Cavity	31 (42)	71 (38)	0.876 (0.550–1.397)	0.579
Bilateral shadow	56 (76)	127 (68)	1.330 (0.781–2.265)	0.294
Pleural effusion	36 (49)	70 (37)	1.503 (0.950–2.380)	0.082

Data are presented as the number (%) or median (interquartile range)

*ALT* alanine transaminase, *AST* aspartate aminotransferase, *BUN* blood urea nitrogen, *COPD* chronic obstructive pulmonary disease, *WBC* white blood cell

Because in the current study we did not follow up patients after discharge, treatment duration in total was unknown. We observed temporal suspension of anti-TB treatment due to adverse effects in 11 patients during hospitalization, and in all patients discharged after confirmation of negative conversion (classified as survivors). The duration of anti-TB drug suspension was 6 d in median (range 2–12 days). The observation period (hospitalization period) for survivors was significantly shorter than that in non-survivors (median 61, IQR 29–119 in survivors vs. 109, 71–152 in non-survivors, p = 0.020).

Patients in the non-survivor group were significantly older (p = 0.002), and had more underlying diseases such as heart failure (p = 0.037), hepatic disease (p = 0.004), chronic kidney disease (CKD) (p < 0.001), respiratory failure on admission (p < 0.001), poorer PS score (p < 0.001), lower albumin level (p < 0.001), and higher C-reactive protein (CRP) level (p = 0.001) than patients in the survivor group.

**Fig. 2:**
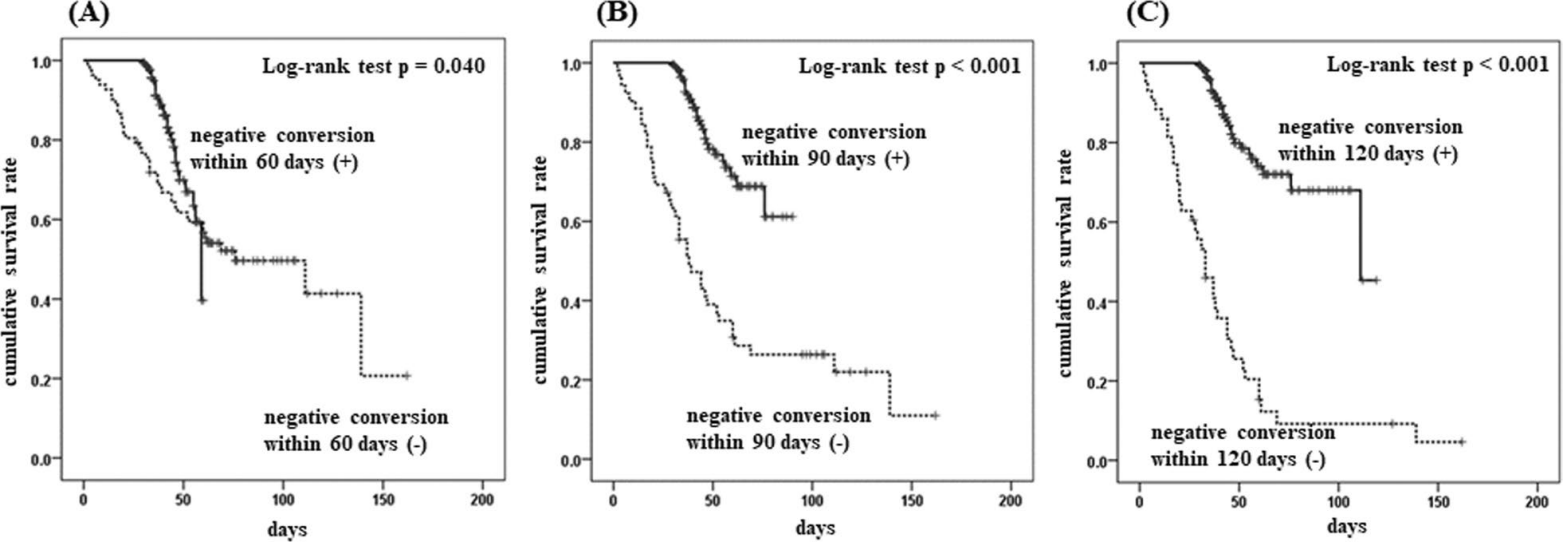
Kaplan–Meier survival probability based on negative sputum culture conversion within 60 days (**A**), 90 days (**B**), and 120 days (**C**) with results from log-rank tests among elderly patients with pulmonary tuberculosis

The conversion rates within 60 days (p = 0.043), 90 days (p < 0.001), and 120 days (p < 0.001) were significantly lower in the non-survivor group than those in the survivor group (Table 1; Fig. 2). For most patients, we regularly tested sputum culture at 2-week intervals after the start of treatment, and we observed no differences in the sampling schedule between non-survivors and survivors, as shown in Table 1. In subgroup analyses, focusing only on patients treated with standard regimens (n = 142), the conversion rates within 90 days (crude hazard ratio 0.147, 95% CI 0.065–0.333, p < 0.001) and 120 days (0.074, 0.033–0.166, p < 0.001) were significantly lower in the non-survivor group than those in the survivor group, whereas the conversion rates for these groups within 60 days did not differ significantly (crude hazard ratio 0.540, 95% CI 0.227–1.287, p = 0.164).

### Predictors of in-hospital mortality in elderly patients with pulmonary tuberculosis

We conducted multivariate analyses using three models because significant differences were observed in sputum conversion within 60 days, 90 days, and 120 days in the univariate analysis adjusting for age, PS, heart failure, CKD, hepatic disease, respiratory failure, standard therapy, serum levels of albumin and CRP, and number of lobes. These analyses showed that sputum non-conversion within 90 and 120 days were independently associated with in-hospital mortality, whereas that within 60 days was not (p = 0.890) (Table 2). Heart failure, hepatic disease, respiratory failure, standard therapy, and number of lobes were not significantly associated with in-hospital mortality in these multivariate models.

**Table 2:** Multivariate analysis of the baseline characteristics associated with the in-hospital mortality of patients with pulmonary tuberculosis

	Model 1 (within 60 days)		Model 2 (within 90 days)		Model 3 (within 120 days)	
	Adjusted hazard ratio (95% CI)	*P*	Adjusted hazard ratio (95% CI)	*P*	Adjusted hazard ratio (95% CI)	*P*
Age, years	1.050 (1.007–1.094)	0.021	1.044 (1.005–1.086)	0.028	1.040 (1.001–1.081)	0.047
Performance status	1.872 (1.213–2.890)	0.005	1.611 (1.029–2.520)	0.037	1.757 (1.136–2.719)	0.011
Chronic kidney disease	3.125 (1.746–5.593)	< 0.001	3.389 (1.811–6.343)	< 0.001	2.499 (1.362–4.587)	0.003
C-reactive protein (mg/dL)	0.959 (0.925–0.995)	0.025	0.939 (0.904–0.975)	0.001	0.955 (0.919–0.994)	0.023
Albumin (g/dL)	0.161 (0.085–0.307)	< 0.001	0.194 (0.101–0.372)	< 0.001	0.212 (0.109–0.412)	< 0.001
Conversion within 60 days	n.s.	n.s.	n.a.	n.a.	n.a.	n.a.
Conversion within 90 days	n.a.	n.a.	0.424 (0.252–0.712)	0.001	n.a.	n.a.
Conversion within 120 days	n.a.	n.a.	n.a.	n.a.	0.333 (0.195–0.570)	< 0.001

These analyses were conducted by adjusting for age, performance status, heart failure, chronic kidney disease, hepatic disease, respiratory failure, standard therapy, C-reactive protein, albumin, and number of lobes

*n.a.* not applicable, *n.s. *not significant

## Discussion

This study showed that the sputum non-conversion within 90 days and 120 days was independently associated with in-hospital mortality. Non-conversion of sputum within 2 months is widely recognized as a poor prognostic factor in patients with pulmonary TB [7–11]. However, we previously reported that in elderly patients with pulmonary TB, sputum conversion within 2 months did not contribute to the prognosis [17]. This study showed that sputum non-conversion within 90 and 120 days, not within 2 months, was associated with in-hospital mortality. These results suggest that 2 months might be insufficient when evaluating sputum conversion as a prognostic factor in elderly patients.

It is important to identify why a longer interval to negative conversion is a predictive factor in elderly patients. First, age-related decline in the immune function may have affected the results. Immune protection against TB infection is primarily achieved by cell-mediated immunity through the coordinated action of phagocytic cells and T cells [25]. However, advanced age reduces T cell output by the thymus, which is known as T cell immune senescence and is defined as the reduced capacity for cell proliferation. Advanced ages also lead to immune exhaustion, defined as the reduced capacity to produce cytokines and other effector molecules [26, 27]. These changes seem to delay the elimination of *M. tuberculosis* and prolong the time to sputum conversion. Second, elderly patients with pulmonary TB mostly have structural changes in their lungs, such as emphysema and bronchiectasis [21], which may decrease *M. tuberculosis* clearance in the lower respiratory tracts. Finally, first-line anti-TB drugs are not well tolerated by elderly patients due to comorbidities [28]. Thus, reduction in drug dosage or discontinuation of treatment is a consideration in these patients [29]. The deviation from standard therapy is expected to prolong the time for elimination of *M. tuberculosis*. However, in the current study, while standard therapy was associated with in-hospital mortality in univariate analysis, it did not reach statistical significance in multivariate analysis. Considering that underlying diseases such as CKD and serum level of albumin were independently related to in-hospital mortality, treatment with standard regimens may be cofounded by these host factors or nutritional status.

CKD is a well-known poor prognostic factor for pulmonary TB [2, 3, 30]. Patients with CKD who develop pulmonary TB have lower treatment success rate and higher mortality [31], which may be explained by inadequate chemotherapy resulting from dose adjustment by renal function. Similarly, the association of low serum albumin and high serum CRP with in-hospital death is consistent with previous studies [19, 32]. While low albumin level reflects poor nutritional status, high CRP represents severe inflammation.

To our knowledge, this is the first study to assess how a long interval to sputum conversion accurately predicts the prognosis in elderly patients with pulmonary TB. These results should be informative not only for countries that have a large number of elderly patients with pulmonary TB, but also for those where the number of elderly patients is expected to increase.

This study has several limitations. First, this was a single-center study with a small number of patients. Second, although this study included elderly patients aged 65 years or older, the median age was much older (84 years). Thus, the results could have been biased by the super-aged population and might be difficult to apply to “general” elderly patients. Third, because of the retrospective nature of this study, there were some missing data such as detailed smoking history. Finally, the primary outcome in this study was all-cause in-hospital mortality. Distinguishing TB-related mortality and non-TB-related mortality would be necessary to accurately evaluate the association between sputum conversion and mortality. Nevertheless, it is challenging to determine whether the mortality was associated with TB or non-TB causes in clinical practice, especially in elderly patients. A multicenter prospective cohort study is required to overcome these limitations of our study.

## Conclusions

Sputum non-conversion was associated with a poor prognosis in elderly patients with pulmonary TB. A period of 2 months after the start of treatment, which is widely regarded as a prognostic factor in the general population, seems insufficient in elderly patients; 90 days or longer appears to allow a more accurate determination of the prognosis. Physicians need to have a longer-term perspective when evaluating therapeutic effect and prognosis in elderly patients with pulmonary TB.

## Data Availability

The datasets analyzed during the current study available from the corresponding author on reasonable request.
